# Life Entrapped in a Network of Atavistic Attractors: How to Find a Rescue

**DOI:** 10.3390/ijms23074017

**Published:** 2022-04-05

**Authors:** Andrzej Kasperski

**Affiliations:** Institute of Biological Sciences, Department of Biotechnology, Laboratory of Bioinformatics and Control of Bioprocesses, University of Zielona Góra, ul. Szafrana 1, 65-516 Zielona Góra, Poland; A.Kasperski@wnb.uz.zgora.pl

**Keywords:** aneuploidy, cancer genome instability, cancer transformation, Crabtree effect, genome chaos, mtNADH molecules, NADH molecule accumulation, unified cell bioenergetics, vertical and horizontal cancer development, Warburg effect

## Abstract

In view of unified cell bioenergetics, cell bioenergetic problems related to cell overenergization can cause excessive disturbances in current cell fate and, as a result, lead to a change of cell-fate. At the onset of the problem, cell overenergization of multicellular organisms (especially overenergization of mitochondria) is solved inter alia by activation and then stimulation of the reversible Crabtree effect by cells. Unfortunately, this apparently good solution can also lead to a much bigger problem when, despite the activation of the Crabtree effect, cell overenergization persists for a long time. In such a case, cancer transformation, along with the Warburg effect, may occur to further reduce or stop the charging of mitochondria by high-energy molecules. Understanding the phenomena of cancer transformation and cancer development has become a real challenge for humanity. To date, many models have been developed to understand cancer-related mechanisms. Nowadays, combining all these models into one coherent universal model of cancer transformation and development can be considered a new challenge. In this light, the aim of this article is to present such a potentially universal model supported by a proposed new model of cellular functionality evolution. The methods of fighting cancer resulting from unified cell bioenergetics and the two presented models are also considered.

## 1. Introduction

The war on cancer was announced in 1971 by then President of the USA, Richard Nixon, and continues today [1]. Although huge amounts of money have been spent and much has been done for this purpose, it is still not enough to empty hospital corridors of people waiting for rescue. Although a lot of research has been conducted, a lot of experimental data has been collected and a lot of articles have been published, it is still not enough to fully understand the mechanism of cancer initiation and progression. There are still phenomena during cancer development that are incomprehensible and therefore considered paradoxical [2]. What do we have today? Today, i.e., after 50 years, the war on cancer goes on, seemingly endlessly [3,4]. Today, a lot of cancer theories have been established (for example, more than 20 theories are presented in [5]), and new theories are introduced all the time, for example, the detached pericyte hypothesis [5]. Pursuant to another interesting idea, the main driver of cancer development is repeated loss of synchronization between the circadian clock (CC) and the cell cycle [6]. This loss of synchronization leads to arrest of the mitotic cell cycle, reprogramming, polyploidization and activation of the ploidy cycle [6]. Then, through depolyploidization, Hayflick’s limit is renewed, cells regain synchronization between CC, and the cell cycle and mitotic cell cycle are activated [6]. Other studies also indicate the importance of polyploidy during cancer development [7,8]. To answer to the question, “what is the reason for cancer, and what is the mechanism of its development”, it is necessary to combine existing theories into one unified theory on the basis of which it will be possible to interpret all phenomena (including that the occurrence of which is considered paradoxical) observed during cancer transformation and development. Most of these theories have been developed to explain phenomena that cannot be explained using the somatic mutation theory (SMT). SMT has been the dominant theory in the study of carcinogenesis for at least sixty years and says that cancer arises from a succession of driver mutations and clonal expansions [9,10]. These phenomena that cannot be explained using SMT (thus proving the weaknesses of SMT) are, among others, (a) causation of cancers by a chemical not known to damage genes, (b) lack of an inducing mutation (for example, in transgenic mouse tumors), (c) infectious causation of cancers and (d) regression of cancer to a benign tumor [11].

The reversal of cancer cells towards an atavistic form of life was formulated by some authors as the atavistic theory of cancer [12,13,14,15,16,17,18,19,20,21,22,23]. It is well known that atavisms can occur experimentally without a mutational basis [24]. This feature of atavisms is especially important in light of the presented weaknesses of SMT and developing new models. Developed atavistic models take into account that cancer onset is a kind of reversion to the quasi-unicellular ancestral phenotype [24]. Evolution of life on Earth over a period of 4 billion years can be divided into two main subperiods. The first and longest subperiod of evolution was dominated by establishing mechanisms responsible for life of unicellular organisms. These mechanisms include mechanisms related to competition, survival and proliferation. Prokaryotic cells probably transitioned into unicellular eukaryotic cells between 2.0 and 1.4 billion years ago [25]. This transition was the basis for the next transition, i.e., the transition to multicellularity. The transition to multicellularity followed the first subperiod and occurred between 1.5 and 0.5 billion years before the present era. During this second, shorter subperiod of evolution, the newer mechanisms (including the pathways) to support multicellular life (inter alia, cell collaboration and differentiation) were established. In accordance with the atavistic cancer model, these newer mechanisms, because they were less well established during the shorter evolution subperiod, are more susceptible to damage than the conservative mechanisms responsible for unicellular life [26]. Damage to the mechanisms responsible for multicellular life can cause cancer transformation. Cancer transformation, according to atavistic cancer models, involves the switch (i.e., the transition) to atavistic unicellular life. This switch is characterized by the occurrence of the Warburg effect and activation of the atavistic mechanisms of competition, survival and proliferation typical of primitive unicellular organisms. A recently proposed exemplary atavistic model assumes that cancer onset and development can be described as a series of reversionary transitions. In accordance with this model (called the serial atavism model), cancer can be considered as not a single atavism with a multicellular-to-unicellular switch but as a sequence of atavistic reversions [24]. Activation of atavistic mechanisms implies the activation of processes that belong to the most conserved and protected processes supported by a myriad of built-in redundant pathways [26]. This may explain the metabolic flexibility in cancer cells that are able to upregulate their compensatory pathways following inhibition of the dominant metabolic pathway [27]. This metabolic flexibility leads to the development of resistance to metabolic inhibitors and is therefore a real challenge in the treatment of cancer [27]. Many attempts have been made to date to eliminate cancer cells, including chemotherapy, hormone therapy, hyperthermia, immunotherapy, photodynamic therapy, radiation therapy, stem cell transplant, surgery and targeted therapy. Striving to understand the universal mechanisms of cancer transformation and development is crucial, as these mechanisms should reveal new ways to eliminate cancer cells more effectively.

This article is organized as follows: firstly, unified cell bioenergetics is presented,, followed by a description of a layered model of evolution of cellular functionalities. Then, the effects of disturbances in cell bioenergetics, along with a universal model of cancer transformation and development (presented for the first time in [28]), among others, are depicted, analyzed and discussed from the perspective of the proposed layered model of evolution of cellular functionalities. Lastly, the research conclusions, together with possibilities of fighting cancer, are presented from the perspective of unified cell bioenergetics and the two presented models (i.e., the layered model of evolution of cellular functionalities and universal model of cancer transformation and development).

## 2. Theoretical Bases

In this section, unified cell bioenergetics and a layered model of evolution of cellular functionalities are presented, constituting the basis for the formulation of a universal model of cancer transformation and development.

### 2.1. Molecular Fundamentals of Unified Cell Bioenergetics and Bioenergetic Disturbances

The phenomenon of cancer can be observed, studied and interpreted both from “the outside” and “the inside”. It is difficult to say which approach is better, although it should be noted that the conclusions drawn from both perspectives must be consistent.

Looking from “the outside”, a very large, genetically heterogeneous set of cells is usually visible [29]. It is known that population genetics meets cancer genomics [30]. The understanding of some population genetic aspects of cancer development can be supported by advances in molecular genetics [30]. Moreover, interpretations of the mechanisms responsible for cancer development and the genetic heterogeneity of cancer cells can be made, for example, by generating phylogenetic trees and analyzing the length of phylogenetic tree branches. Observations from these analyses show that mutations accrue faster in some cancer regions than in others, as evidenced by the large variations in branch length [29]. Interesting conclusions from this observation include that a malignant tumor (i.e., colorectal cancer as an exemplary examined malignant tumor) occupies a sharper fitness peak compared to that of a benign tumor (i.e., adenoma as an examined exemplary benign tumor) which evolves across an undulating fitness landscape [29].

From “the inside”, i.e., from the perspective of the low-molecular level, a lot of different phenomena result from a huge number of metabolic pathways. For this reason, at first, it is important to select fundamental phenomena that can be considered as main bioenergetic drivers of life. The next attempt should be to combine these fundamental phenomena into one coherent whole in order to understand the universal bioenergetic mechanisms that drive life. Then, from the perspective of this unification, one can try to interpret more and more complex phenomena step by step. In this article, the second attempt is presented, i.e., studies and interpretations of cancer phenomena from “the inside”.

The fundamental cellular phenomena that can occur during eukaryotic cell metabolism include the Pasteur, Crabtree, Kluyver and glucose effects. However, by examining the occurrence of each of these effects individually, it is extremely difficult to understand the more complex mechanisms that drive the life of multicellular organisms. A solution to take a step forward in understanding might be to unify all these fundamental bioenergetic effects into one generalized effect. According to unified cell bioenergetics (UCB), the unification of these fundamental effects is possible by examining the intramitochondrial level of energy-storing NADH molecules (i.e., mtNADH molecules) [31,32]. In the eukaryotic cells, the Krebs cycle rotation is coupled with several reactions (i.e., reductions), from NAD to NADH [33]. In accordance with UCB, mitochondria are charged with NADH molecules during the Krebs cycle, and NADH is discharged from mitochondria in the electron transport chain [28,31,32,34,35]. In this light, overloading the cell with an excessive amount of food or malfunctioning oxidative phosphorylation (OXPHOS) can lead to serious bioenergetic problems related to the accumulation of huge amounts of high-energy molecules (especially NADH) in mitochondria [31,32]. The accumulation of high-energy molecules, as a result overenergization of mitochondria, may occur due to the impermeability of the mitochondrial inner membrane to NADH [36,37]. NADH accumulation occurs (as a consequence of the impermeability of the mitochondrial inner membrane to NADH) when the rate of charging mitochondria with NADH is higher than the rate of discharging mitochondria from NADH [32]. Under these conditions, a gradual increase in NADH occurs. An increase in NADH causes an exponential increase in ROS [38,39,40]. A high ROS level poses an additional problem for the cell, as it can result in severe oxidative damage to DNA, biolipid membranes and proteins [3,41,42]. In order to protect mitochondria from overenergization, the cell stimulates aerobic fermentation. The occurrence of fermentation under good aerobic conditions is called the Crabtree effect [32,43]. In light of unified cell bioenergetics, the Crabtree effect occurs when the intramitochondrial NADH amount exceeds the mtNADH_normal_ level (i.e., the normal level of intramitochondrial NADH) [32]. The Crabtree effect precedes the occurrence of the Warburg effect [31,44,45]. In accordance with unified cell bioenergetics, bioenergetic cell problems, especially overenergization of mitochondria by NADH molecules, can lead to cancer transformation and subsequent cancer development [28,31]. Moreover, cancer cells remain overenergized after transformation and during cancer development (see Section 3.5) [28,31]. For this reason, cancer cells also have higher levels of ROS than their non-cancerous cells of origin. This conclusion was also confirmed by other researchers [42,46,47,48,49,50]. Moreover, it is known that excessive levels of ROS cause cell death; therefore, cancer cells adapt multiple metabolic strategies (by adaptation of genome expression) to avoid excessive increases in ROS beyond the level that causes excessive oxidative stress and leads to cell death [46].

### 2.2. Layered Model of Evolution of Cellular Functionalities

During the process of building complex and functional multicellular organisms, strong constraints preventing inappropriate atavisms (including uncontrolled cell proliferation) also had to evolve [51]. This process did not necessarily remove the ancestral programs but rather prevented their expression [52]. Multicellular programs (that evolved during this process) are believed to be oncosuppressive, and when working properly, they control unicellular programs [53]. For this reason, malfunctions in the operation of multicellular programs due to cancer-promoting factors may cause the activation of unicellular-like programs that promote cancer [53]. According to the systemic–evolutionary theory of cancer (i.e., SETOC, one of the newest theories about the origin of cancer), cancer is generated by the re-emergence of older cellular evolutionary subsystems (such as archaea-like and/or prokaryotic-like subsystems) that are characterized by uncoordinated behaviors [53]. The possibility of activation of older, ancestral (primordial) evolutionary functionalities (i.e., according to the SETOC: unicellular-like programs) by cells of multicellular organism suggests that during evolution, older functionalities are stored, undergoing activity control by newer evolutionary functionalities (i.e., according to the SETOC: multicellular programs). These atavistic (or primordial) behaviors are preserved by cells as a phylogenetic memory [53]. A phylogenetic memory is represented in a cell by a genome [54]. Repair mechanisms enable this memory to have a very low error rate (i.e., 1 in 10^10^ base pairs), which is incredibly accurate [54]. For this reason, the stability of the phylogenetic memory that accumulates knowledge for millions of generations is very high [54]. This indicates that during evolution, a hierarchical organization of cellular functionalities is established with very precisely stored and preserved ancestral functionalities.

In this work, a new model of evolution of cellular functionalities is proposed. In accordance with this model, cellular functionalities are located in layers, where new, more developed cellular functionalities are added to the more external layers. In this way, during the evolution, a layered structure of functionalities is created in which the outer layers contain new functionalities that can use, control (including deactivation) and extend the old functionalities that are stored (as a phylogenetic memory) in more internal layers. For example, the functionality of facultative switching between oxidative phosphorylation and aerobic glycolysis extends the less developed functionality of aerobic glycolysis [55]. Huge disturbances (or destruction) of functionalities can lead to a loss of control over functionalities of more internal (i.e., atavistic) layers, i.e., such disturbances can lead to an uncontrolled reactivation of an evolutionarily older cell fate.

#### 2.2.1. Layer of Cell Bioenergetic Functionalities, i.e., a Phylogenetic Memory Layer of Universal Cell Bioenergetic Functionalities

**Figure 1:**
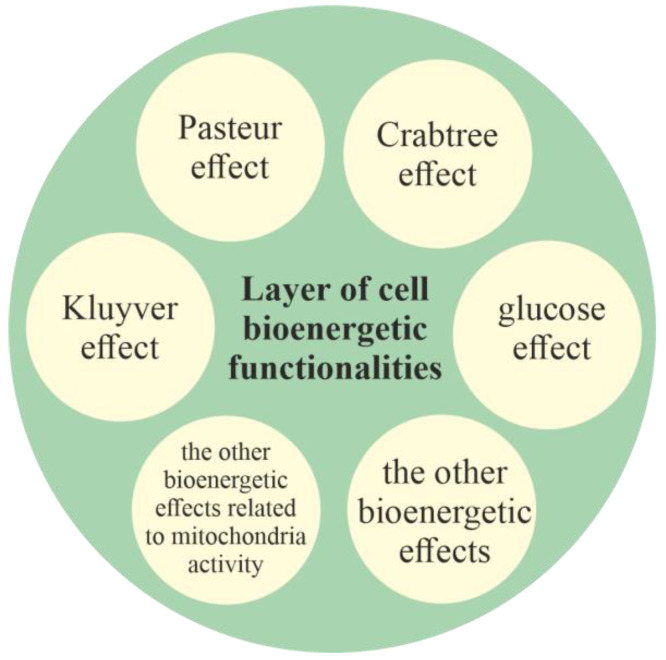
The layer of cell bioenergetic functionalities (i.e., the cell bioenergetic layer) contains fundamental functionalities that can be considered main bioenergetic drivers of cell life.

The layer of cell bioenergetic functionalities, as layer that contains functionalities of main bioenergetic engines of cell life and development, is localized in the innermost part of the model (Figure 1). The layer of cell bioenergetic functionalities includes the bioenergetic functionalities needed for life, including, functionalities responsible, inter alia, for the occurrence of the Pasteur, Crabtree, Kluyver and glucose effects (i.e., functionalities that constitute the functionalities of unified cell bioenergetics). Is should be noted that in accordance with the endosymbiotic theory, mitochondria are the early prokaryotic endosymbionts that entered a larger cell about 1.5 billion years ago (i.e., during the process of transition of prokaryotic cells into unicellular eukaryotic cells) [56]. Because mitochondria constitute the main powerhouses the of cells, the other bioenergetic effects related to mitochondrial activity are part of the layer of cell bioenergetic functionalities.

#### 2.2.2. Layer of Unicellular Functionalities, i.e., a Phylogenetic Memory Layer of Atavistic Functionalities

**Figure 2:**
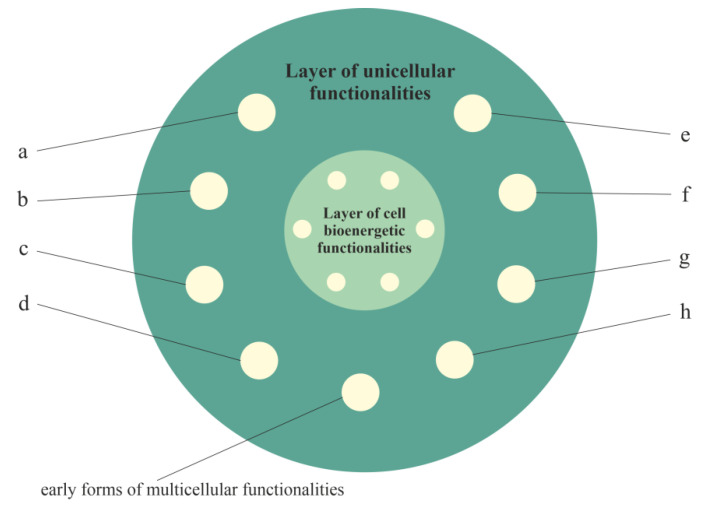
The layer of unicellular functionalities (i.e., the unicellular layer) inherits from the layer of cell bioenergetic functionalities, i.e., functionalities of the second layer can use, control (including deactivation) and extend inherited functionalities. Selected functionalities presented in this figure of the unicellular layer include (a) unregulated, rapid and aggressive angiogenesis; (b) unregulated cell proliferation; (c) a lack of a Hayflick’s limit; (d) relief from curfew and checkpoints; (e) wound healing that does not stop stem-cell-like behavior; (f) unregulated EMT migration, aggressive invasion and metastasis; (g) aerobic glycolysis; and (h) unregulated and truncated cell-differentiation cascades.

The activity of functionalities of the cell bioenergetic layer has to be controlled (i.e., integrated and synchronized by appropriate stimulation and inhibition) to meet the cell’s bioenergetic requirements. According to the proposed model, during evolution of unicellular organisms, functionalities of the unicellular layer were formed that used, controlled and extended the functionalities of the cell bioenergetic layer (Figure 2). This layer (i.e., the layer of unicellular functionalities) provides basic life functionalities, including competition, survival and proliferation of unicellular organisms. These functionalities belong to the most conserved and protected functionalities supported by a myriad of built-in redundant pathways [26]. The plethora of built-in redundant pathways also indicates that these functionalities are very elastics and adaptive, i.e., they can remain active in a heavily altered environment, despite changes in the genome.

In accordance with the information presented in Table 1 in [55], the other functionalities of the unicellular layer are related, among other factors, to (a) unregulated, rapid and aggressive angiogenesis; (b) unregulated cell proliferation; (c) the lack of a Hayflick’s limit; (d) relief from curfew and checkpoints; (e) wound healing that does not stop stem-cell-like behavior; (f) unregulated epithelial–mesenchymal transition (EMT) migration; aggressive invasion and metastasis; (g) aerobic glycolysis; and (h) unregulated and truncated cell-differentiation cascades.

It should be added that many of the developmental requirements for multicellular organization (including functionalities of cell adhesion, cell–cell communication and coordination, and programmed cell death) probably existed in ancestral unicellular organisms [57]. For this reason, these functionalities are also located in the unicellular layer of the model and are presented in Figure 2 as early forms of multicellular functionalities.

#### 2.2.3. Layer of Multicellular Functionalities, i.e., a Phylogenetic Memory Layer of Multicellular Advanced Functionalities

**Figure 3:**
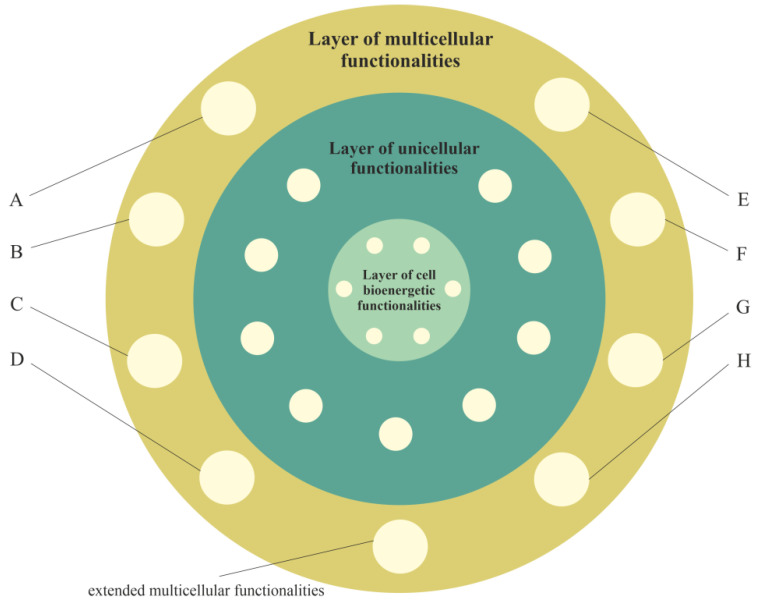
The layer of multicellular functionalities (i.e., multicellular layer) directly inherits from the layer of unicellular functionalities and indirectly from the layer of bioenergetic functionalities, i.e., functionalities of the third layer can use, control (including deactivation) and extend inherited functionalities. Selected functionalities of the multicellular layer include (A) normal well-regulated angiogenesis; (B) well-regulated cell proliferation, along with signaling, to control proliferation; (C) Hayflick’s limit and functionality of p53; (D) cell cycle checkpoints; (E) signaling bringing to an end wound healing; (F) regulated release; (G) facultative switching between oxidative phosphorylation and aerobic glycolysis; and (H) normal, well-regulated cell differentiation.

The evolution of multicellularity required the integration of single cells into new functionally, reproductively and evolutionary stable multicellular organisms [58]. This process required new functionalities. According to the proposed model, during evolution of multicellular organisms, functionalities of the new layer were formed that could use, control and extend both the functionalities of the layer of unicellular functionalities and layer of cell bioenergetic functionalities (Figure 3). This layer (i.e., the layer of multicellular functionalities) provides complex life functionalities to multicellular organisms, including extended functionalities of proliferation, cell–cell communication, coordination, integration, adhesion to neighboring cells, signaling to maintain adhesion and programmed cell death. These functionalities are presented in Figure 3 as extended multicellular functionalities.

In accordance with the information presented in Table 1 in [55], the other functionalities of the multicellular layer (that hide/control functionalities of the unicellular layer) are related, among other factors, to (hidden/controlled functionalities are written in square brackets): (A) normal well-regulated angiogenesis [unregulated, rapid and aggressive angiogenesis]; (B) well-regulated cell proliferation, along with signaling, to control proliferation [unregulated cell proliferation]; (C) Hayflick’s limit and functionality of p53 [no Hayflick’s limit]; (D) cell cycle checkpoints [relief from curfew and checkpoints]; (E) signaling bringing to an end wound healing [wound healing that does not stop stem-cell-like behavior]; (F) regulated release [unregulated epithelial–mesenchymal transition (EMT) migration, aggressive invasion and metastasis]; (G) facultative switching between oxidative phosphorylation and aerobic glycolysis [aerobic glycolysis]; and (H) normal, well-regulated cell differentiation [unregulated and truncated cell-differentiation cascades].

## 3. Discussion on the Universal Model of Cancer Transformation and Development

As a phenomenon, cancer is a disease related to multicellular evolution, i.e., cancer in general is understood to be a failure of the multicellular systems and is considered a reversal to unicellularity [58,59]. Cancer cells are like unicellular organisms that benefit from ancestral-like traits [58]. As a disease, cancer can be interpreted as (a) a destruction of cooperative behaviors underlying multicellular evolution, (b) a disruption of molecular networks established during the emergence of multicellularity or (c) an atavistic state resulting from reactivation of primitive programs typical of the earliest unicellular species [58]. From this point of view and in accordance with the layered model of evolution of cellular functionalities, cancer transformation can occur as a result of huge disturbances or the destruction of functionalities that are located in the multicellular layer. In this light, the universal model of cancer transformation and development can be considered an extended and improved model in comparison with those presented in previous articles [28,31].

### 3.1. Cancer Transformation as a Loss of Control over Atavistic Functionalities

**Figure 4:**
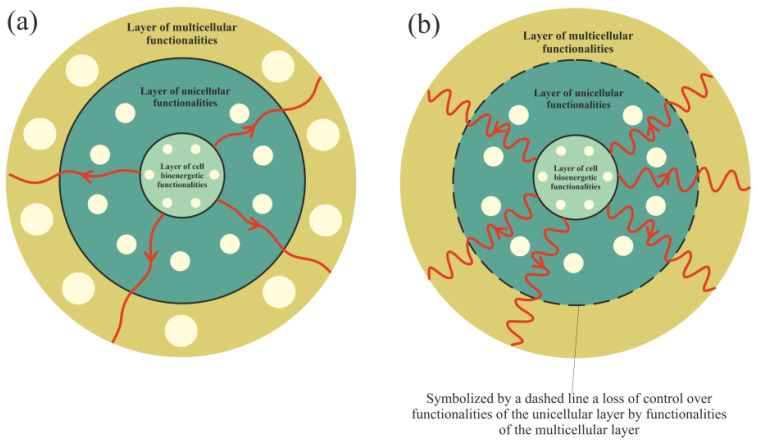
Propagation of disturbances in cell bioenergetics. (**a**) Small disturbances cause small disturbances in functionalities of the unicellular and multicellular layers. (**b**) Huge disturbances can cause a loss of functionalities of the multicellular layer (or huge disturbances in their operation), leading to a loss of control over unicellular layer functionalities.

Functionalities of the multicellular layer are located in the most external layer of the layered model of evolution of cellular functionalities. In accordance with unified cell bioenergetics, bioenergetic cell problems, especially overenergization of mitochondria, can lead to cancer transformation [28,31]. Cell overenergization (and the related increase in ROS) is followed by adaptation of multiple metabolic strategies to solve this bioenergetic problem [46]. As a result, in light of the layered model of evolution of cellular functionalities, the cell’s response to a huge bioenergetic problem related to overenergization is propagation of disturbances in genome expression from the most internal (i.e., from the layer of bioenergetic functionalities) toward the more external layer (i.e., toward the layer of multicellular functionalities). Functionalities that are localized in the layer of multicellular functionalities, as the most complex and evolutionarily youngest (see Introduction), are the most sensitive to disturbances. The disturbance (or destruction, for example by, high ROS levels) of multicellular layer functionalities can result in a loss of control over functionalities of the unicellular layer, leading to cancer transformation, i.e., uncontrolled activity of atavistic functionalities (Figure 4).

### 3.2. Vertical and Horizontal Cancer Development

**Figure 5:**
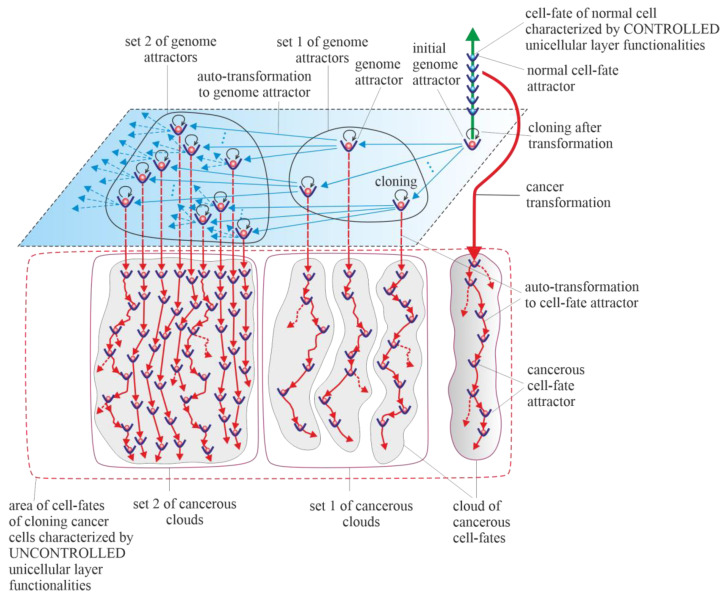
Schematic view of the universal model of cancer transformation and development presented from the perspective of the layered model of evolution of cellular functionalities (especially from the perspective of losing control over functionalities of the unicellular layer). After cancer transformation, cancer development occurs as the development of a population of individual cloning cells. Moreover, cancer development shows attractor-like behavior, i.e., vertical cancer development occurs through step-by-step changes of cell-fate attractors, and optional horizontal cancer development occurs through step-by-step changes in genome attractors.

Results of studies on tumor development published to date (deep sequencing, multi-region sequencing and single-cell sequencing) indicate that a single normal cell is a common origin of cancer [60]. In order to increase the probability of survival, cancer transformation initiates the creation of multiple clones, along with activation of the ploidy cycle (see Introduction) [6,61]. Transformation also initiates cancer development, which, according to the universal model of cancer transformation and development (Figure 5), occurs as the development of a population of individual cloning cells. Due to the very high complexity of the task, establishing a universal model of cancer transformation and development can be treated as a process of successive extensions and improvements. The first approaches to establishing such a universal model were presented in previously published articles [28,31]. In this work, the universal model of cancer transformation and development (Figure 5) is presented from the perspective of the proposed layered model of evolution of cellular functionalities (especially from the perspective of losing control over functionalities of the unicellular layer), which can be considered a significant extension and improvement compared to the previously presented approaches. In this context, Figure 5, with a very synthetic explanation (compared to that previously presented in Figure 7 in [28] and Figure 3 in [31]) allows for a more complete understanding of the presented considerations. According to the universal model of cancer transformation and development, vertical and optional horizontal development can be distinguished [28]. Both vertical and horizontal cancer development occur through step-by-step changes of attractors, i.e., caner development shows attractor-like behavior. The idea of “cancer attractors” was first proposed by Stuart Kaufman and has been supported experimentally [62,63]. The use of the attractor idea has made it possible to explain why the cells of multicellular organisms are prone to oncogenesis [64]. In general, the application of the attractor concept allows the physical concept to be used in biological reality, where the term “attractor” denotes the configuration towards which the system evolves over time to achieve stability [65,66]. Attaining an attractor means that a given system configuration is stable enough to return to its original state after the disappearance of any small disturbances [66]. Additionally, in Figure 5, exemplary attractors of normal cell fates are marked on the vertical green axis. It should be noted that all normal cell fates are generated by cells trapped in one genome attractor. This is because genome DNA sequences in each cell nucleus are identical in human cells (and tissues of each individual) [67].

### 3.3. Vertical Cancer Development

Vertical cancer development occurs when cells change cell-fate attractors without a change in genome attractor, and for this reason, this type of cancer development can be considered a kind of microevolution. A change in cell-fate attractor can occur as a result of the occurrence of considerable instability of genome expression (i.e., considerable instability of current cell fate) [68,69]. Vertical cancer development can occur without mutations (for example, only as a result of cell bioenergetic problems) or with mutations as an associated phenomenon (but under the condition that these mutations do not cause leaving of the genome attractor). Autotransformation to cell-fate attractor (i.e., transformation that constitutes an ordered cell response to cell-fate instability) causes ordered changes of genome expression introduced in order to attain cell-fate stability [28]. That means that autotransformation to the cell-fate attractor causes stabilization of cell-fate in the new cell-fate attractor. As has been presented in published articles, activation and stabilization of new cell-fate occurs by positional chromatin remodeling [68,69].

### 3.4. Horizontal Cancer Development

Horizontal cancer development occurs when cells change genome attractors, and for this reason, this type of cancer development can be considered a kind of macroevolution. It should be added horizontal cancer development is optional; it may occur or it may not [28]. Change in genome attractor can occur as a result of a loss of genome stability. Destabilization of the genome can follow destruction of the DNA fragments that code mechanisms responsible for monitoring genomic integrity by random mutations caused by a high level of ROS [70]. The destruction of these mechanisms and consequent loss of genomic integrity can lead to genome instability (GIN) and, as a result, to genome chaos. Genome chaos is a process of complex, rapid genome reorganization that results in the formation of unstable genomes [71]. These unstable genomes have the potential to establish stable genomes [71]. Taking into account that during cancer development, adaptation of clones to stress occurs by the production of new genomes that are essential for phase transition, the occurrence of genome chaos is an important factor in cancer development [1,72,73,74,75]. The aim of autotransformation to the genome attractor (which constitutes an ordered cell response to the formation of an unstable genome as a result of occurrence of genome chaos) is stabilization of unstable genomes [28]. Autotransformation to the genome attractor causes unstable genome changes (including aneuploidy, rearrangements and other ordered genome changes) introduced in order to attain genomic stability. That means that autotransformation to the attractor causes stabilization of the genome in a new genome attractor. Attaining a new genome attractor has to be followed by a change of cell fate in order to keep the cell alive by adjusting genome expression to the changed genome. That means that autotransformation to the attractor has to be followed by autotransformation to the cell-fate attractor [28]. From this point of view, cancer development occurs as a kind of process of self-creation, i.e., “under high-stress conditions likely to eliminate a system, the system’s cellular machinery will automatically switch into a mode that destroys the current genome and simultaneously forms new genomes using their own genomic materials” [1]. The aim of cancer development as a process (including, among other factors, successive losses of genomic integrity that lead to subsequent genome instability (GIN), genome chaos, formation of unstable genomes followed by autotransformation to the genome attractor and autotransformation to the cell-fate attractor) is to form new genomes and keep cells alive.

Cancer is characterized by abundant genetic abnormalities in the form of mutations, single-nucleotide polymorphisms, copy-number alterations, genomic rearrangements and gene fusions [76]. Moreover, aneuploidy appears early during cancer development and correlates with cancer aggression and resistance to anticancer treatments, favoring cancer progression and poor prognosis [6,77,78,79,80,81]. Resistance to anticancer treatments may be related to the possibility of recovering an individual gene’s function by aneuploidy [82,83]. Cancers are known to be clonal for aneuploidy above a certain threshold [84]. Aneuploidy is a phenomenon associated with horizontal cancer development. In accordance with the universal model of cancer transformation and development, horizontal cancer development causes a perpetual increase in aneuploidy, along with permanent cloning, when aneuploidy passes the threshold. A change in the karyotype (i.e., a change in the whole sets of functionalities) related to the addition or removal of one small chromosome can alter overall gene expression [82,83]. That means that aneuploidy has a large impact on cancer development, especially on genome and cell-fate heterogeneity.

Vertical and horizontal cancer development allows the cancer cells to test the genomic landscape. Horizontal development as macroevolution is associated by large changes in the genome and allows for simultaneous testing of the genomic landscape in many evolutionarily distant places (i.e., horizontal development allows for global testing of the genomic landscape). Vertical development as microevolution is associated with development without changes or small changes in the genome that do not cause changes in the genome attractor. Vertical cancer development confirms whether it is possible to keep clones alive (by changing cell fates appropriately) in distant places (established by horizontal development), i.e., in genome attractors. Additionally, vertical development allows clones to adapt to the environment by cell-fate changes. An exact adaptation to environment can occur by local testing of the genomic landscape, i.e., by cloning and small changes in the genome (caused by high ROS levels) and, related to these changes, changes in cell fates. In this way, adjusting of cell fates to extracellular and intracellular conditions can occur.

Horizontal cancer development can be compared to sowing different seeds (seeds represent clones) in a large field. Vertical cancer development can be compared to checking whether it is possible to maintain life in given places in the field and to adjusting life to these places.

The information presented in this article so far can be summarized as follows:

(a) Cancer transformation is caused by a loss of control over atavistic functionalities;
(b) Vertical cancer development is caused by recurring (repeated) losses of current cell-fate stability; at
(c) Horizontal cancer development is optional and is caused by recurring (repeated) losses of genomic integrity.

### 3.5. Cancerous Clouds of Atavistic Cell-Fates

**Figure 6:**
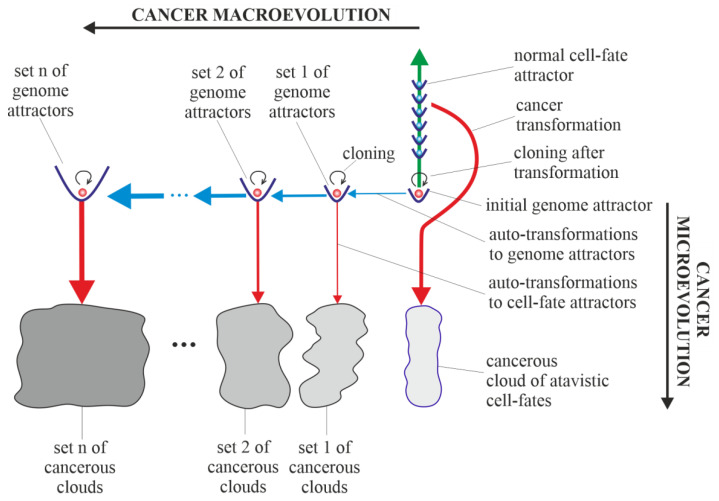
Schematic view of cancer micro- and macroevolution. Sets of cancerous clouds are generated by cells trapped in a network of genome attractors.

The Warburg effect, a phenomenon associated with cancer transformation and development, causes intensive stimulation of fermentation under good aerobic conditions [85,86,87]. Intensive fermentation allows cells to obtain needed energy during glycolysis. The rate of energy attainment after cancer transformation from intensive glycolysis can be higher than the rate of energy attainment from oxidative phosphorylation (OXPHOS). OXPHOS is a very effective way to obtain energy, but taking into account that a possible fermentation rate can be 100 times quicker than the oxidative process of ATP generation by mitochondria, the rate of energy attainment from glycolysis (i.e., in the glycolysis-fermentation pathway) can be about six times higher in comparison to OXPHOS [88,89]. After transformation, the large amount of obtained energy in the glycolysis-fermentation pathway prevents discharge of mitochondria from high-energy molecules [31]. As a result, this phenomenon causes cancer mitochondria to remain overenergized. Overenergization, as an internal cell problem, is a factor that drives cancer vertical development (i.e., cancer microevolution) and cancer horizontal development (i.e., cancer macroevolution) (Figure 6). A large amount of obtained energy after transformation also allows for intensive cell cloning, which creates a network of cell fates (herein termed “cloud of cell-fates”). A cloud of cell fates is generated by cells initially trapped in one (initial) genome attractor. Considering the case of horizontal cancer development, the network of genome attractors undergoes gradual and dynamic expansion. As a result, sets of cancerous clouds (that contain cancerous clouds of atavistic cell fates) are generated by cloning cells trapped in a dynamically emerging and altered network of genome attractors, as schematically shown in Figure 6.

### 3.6. Purely Vertical Cancer Development

**Figure 7:**
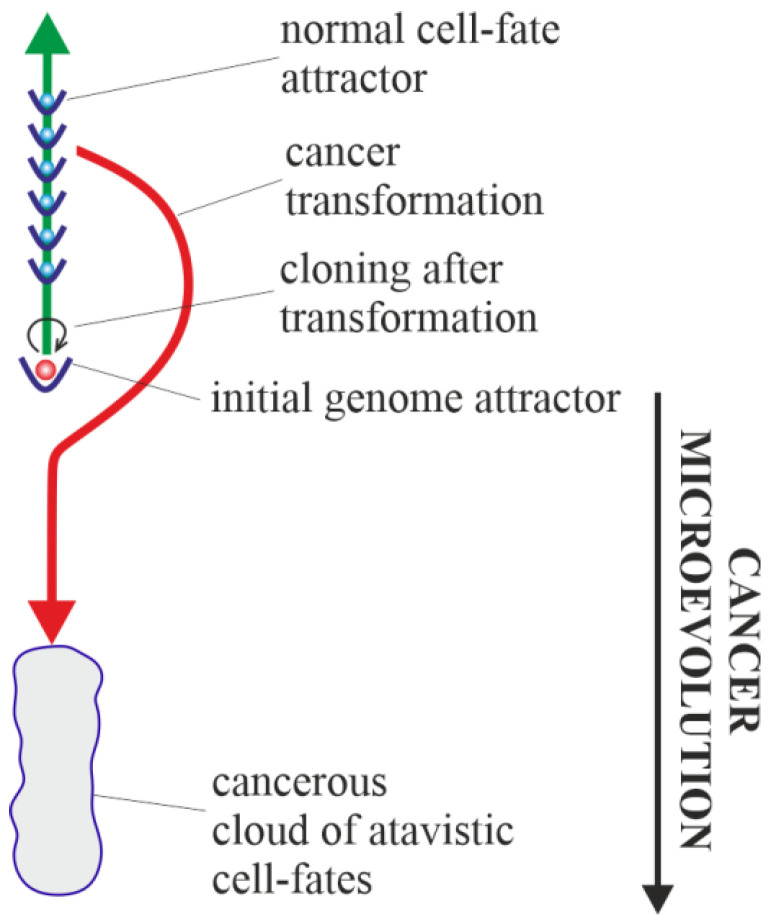
Purely vertical cancer development. After transformation, cancer development occurs only as a microevolution, i.e., only as subsequent changes in cell-fate attractors.

Horizontal cancer development is optional (i.e., it may occur or not). From this point of view, a special case of vertical cancer development is vertical development, with no one change of genome attractor (i.e., purely vertical cancer development). This means that during cancer development, cancer clones are trapped and kept in the initial genome attractor. The initial genome attractor is a genome attractor of a normal cell in which the cell has been transformed into a cancerous cell (see Section 3.2). After transformation, cancer development occurs as subsequent changes in cell-fate attractors due to destabilizations of current cell fates (Figure 7). Destabilization of cell fates can occur as a result of bioenergetic problems (i.e., without mutations) and as a result of mutations that do not cause a change in genome attractor.

### 3.7. Cancers without Mutation

Teratomas and choriocarcinomas, as exemplary cancers without mutations, are formed by misplaced embryonic and placental cells [90]. These cells are characterized by altered expression of hundreds of oncogenes and tumor suppressors (silenced or induced by epigenetic mechanisms) compared to adult tissues [90]. In choriocarcinoma, HLA-G is demonstrated to change the tumor microenvironment through the inactivation of the local immune system at very high levels and functions [91]. Moreover, choriocarcinoma is characterized by overexpression of p53 and MDM2, along with overexpression of other genes (i.e., NECC1, epidermal growth factor receptor, DOC-2/hDab2, Ras GTPase-activating protein, E-cadherin, HIC-1, p16 and TIMP3) or downregulation via hypermethylation, with no evidence of somatic mutation [91]. In this light, a special case of vertical cancer development is purely vertical cancer development without any mutation. That means that during cancer development, cancer clones are trapped and kept in the initial genome attractor, and additionally, cancer development occurs without mutations (i.e., only as a result of bioenergetic problems that lead to subsequent destabilizations of cell fates and changes in cell-fate attractors).

### 3.8. Cancer Development as a Learning Process

Cancer progression can be considered a learning process [92]. This learning process is very costly because a large number of cancerous cells dies during cancer development [93,94,95]. Taking into account that the cancer cell-doubling time is around 1–2 days and that tumor-doubling time is around 60–200 days, the conclusion is that the vast majority of cancer cells die before they can divide [93,94,95]. Even advanced malignancies can exhibit such characteristics of growth (i.e., Gompertzian growth) [94]. In light of the universal model of cancer transformation and development, horizontal cancer development is driven by successive destructions of mechanisms responsible for monitoring genomic integrity. Consequently, a loss of genomic integrity can lead to genome instability (GIN) and, as a result, genome chaos and formation of unstable genomes (as an outcome of genomic chaos). Taking into account cancer Gompertzian growth, in light of the universal model of cancer transformation and development, only a small part of the unstable genomes can undergo transformation (by autotransformation to the genome attractor) to stable genomes and attain stability in genome attractors. It should also be taken into account that not all stable genomes have the potential to keep the cell alive. This means that only part of the clones with stable genomes can undergo successful vertical transformation and attain cell-fate stability in cell-fate attractors.

## 4. Conclusions

The large number of theories related to cancer transformation and development that exist today highlights the need to find a long-awaited recipe for cancer. The considerable complexity and number of phenomena related to cancer transformation and development have resulted in various theories focused on different layers (molecular, cell, tissue, organism, etc.) in order to explain these phenomena. The bioenergetics-focused approach outlined in this article has the potential to unify the existing cancer theories because bioenergetics is “dispersed” in different layers, permeating and affecting each of them.

Considerations presented in this article provide an answer to the question “what is the cause of cancer?”. In light of the presented considerations, the reason for cancer is a change in normal cell fate to cancerous/atavistic cell fate (Figure 5, Figure 6 and Figure 7). In this article, it was proposed that cancer transformation (i.e., a change in normal cell-fate to cancerous/atavistic cell fate) occurs as a result of loss of control over functionalities of the unicellular layer, resulting in a loss of control over atavistic functionalities (Figure 4). This conclusion is also supported by other research, for example, [96]. Cancer transformation can occur as a result of huge disturbances in functionalities of the multicellular layer that normally control activity of atavistic functionalities. This means that cancer transformation can occur without any mutation and only as a result of cell bioenergetic problems. Cancer transformation can also occur with mutations, provided that in a given cellular state, huge disturbances (or destruction) of functionalities of the multicellular layer appear.

According to the universal model of cancer transformation and development, cancer cells exhibit a high heterogeneity due to permanent changes in genome attractors and cell-fate attractors. Because of this heterogeneity and uncontrolled activity of atavistic functionalities (that are conserved, protected and redundant), it is extremely difficult to find a recipe for cancer. In line with unified cell bioenergetics, the layered model of evolution of cellular functionalities and the universal model of cancer transformation and development, the following guidelines can be useful in the fight against cancer:

(a) It is better not to concentrate on destruction of unicellular layer functionalities (for example, aerobic glycolysis, which occurs during the Warburg effect); such functionalities are very conservative and secured by many redundant metabolic pathways;
(b) In order to stop permanent changes of genome attractors, cancerous mitochondria should be discharged from high-energy molecules, among others, by increasing physical activity, intensive aeration and ensuring a proper diet with food limitation. This conclusion is in accordance with the mitochondrial correction method, which represents a new therapeutic paradigm for cancer [97]. The disadvantage of the discharging mitochondria method is that after discharge, driven by uncontrolled atavistic functionalities, cancerous cell fates are most likely still active;
(c) In order to regain control over atavistic functionalities of the unicellular layer, functionalities of the multicellular layer should be reconstructed and reactivated. This idea is related to an old concept of tumor reversion [98]. Studies demonstrated that when tumor cells are placed within a “normal” morphogenetic milieu, they can be “reprogrammed” (thus acquiring a healthy phenotype de novo) and can then behave as native cells [99]. For example, several cancers undergo partial or complete reversion after exposure to embryonic environments [100]. This phenotypic reversion can be achieved despite the large number of genome alterations presented in cancerous cells and is related to inhibition of the migrating/invasive phenotype of cancer cells by some low-molecular-weight factors expressed by early embryonic microenvironments [101,102,103,104]. In this light, one possibilities for reconstruction and reactivation of functionalities of the multicellular layer is to expose cancer to embryo and/or egg extracts [105,106]. The disadvantage of this method is that after reconstruction and reactivation of functionalities of the multicellular layer, mitochondria are most likely still overenergized, which threatens repeated cancer transformation;
(d) The best solution seems to be the discharge of cancerous mitochondria from high-energy molecules, along with the reconstruction and reactivation of functionalities of the multicellular layer.

## Data Availability

Not applicable.
